# Operation of Gastronomy for the Relief of Internal Strangulation

**Published:** 1847-04

**Authors:** 


*Operation of Gastronomy for the Relief of Internal Strangulation.*—We are informed that the operation of gastronomy has been recently performed by Mr. Hilton, of Guy's Hospital, with complete relief to the intestinal obstruction, in a man, twenty years of age, suffering from internal strangulation of a large portion of ileum of twelve days' duration. The point of obstruction was situated about twelve inches from the cæcum. The patient died of exhaustion a few hours after the operation. Full particulars of the case will shortly be given at the Medical and Chirurgical Society.—*Lond. Med. Gaz.*

